# Expression Cloning and Production of Human Heavy-Chain-Only Antibodies from Murine Transgenic Plasma Cells

**DOI:** 10.3389/fimmu.2016.00619

**Published:** 2016-12-19

**Authors:** Dubravka Drabek, Rick Janssens, Ernie de Boer, Rik Rademaker, Johannes Kloess, John Skehel, Frank Grosveld

**Affiliations:** ^1^Department of Cell Biology, Erasmus MC, Rotterdam, Netherlands; ^2^Harbour Antibodies BV, Rotterdam, Netherlands; ^3^WHO Influenza Centre, Frances Crick Institute, London, UK

**Keywords:** plasma cells, transgenic mice, human VH, HCAb, HEK cell library

## Abstract

Several technologies have been developed to isolate human antibodies against different target antigens as a source of potential therapeutics, including hybridoma technology, phage and yeast display systems. For conventional antibodies, this involves either random pairing of VH and variable light (VL) domains in combinatorial display libraries or isolation of cognate pairs of VH and VL domains from human B cells or from transgenic mice carrying human immunoglobulin loci followed by single-cell sorting, single-cell RT-PCR, and bulk cloning of isolated natural VH–VL pairs. Heavy-chain-only antibodies (HCAbs) that naturally occur in camelids require only heavy immunoglobulin chain cloning. Here, we present an automatable novel, high-throughput technology for rapid direct cloning and production of fully human HCAbs from sorted population of transgenic mouse plasma cells carrying a human HCAb locus. Utility of the technique is demonstrated by isolation of diverse sets of sequence unique, soluble, high-affinity influenza A strain X-31 hemagglutinin-specific HCAbs.

## Introduction

*Camelidae* produce not only conventional antibodies, composed of two heavy and two light chains (H2L2), but also antibodies composed of heavy chains only. Although in the conventional antibodies both chains contribute to the antigen binding site, the antigen binding site of camelid heavy-chain-only antibodies (HCAbs) is formed by single heavy chain variable domain (VHH) (1, 2). We have previously generated transgenic mice containing hybrid llama-human antibody loci with two llama variable VHH regions and human D, J, and C_µ_ and/or C_γ_ constant regions. Such loci rearrange productively and rescue B cell development efficiently (3).

Heavy-chain-only antibodies are expressed at high levels in camelids (4) and in transgenic mice (3, 5), provided that the CH1 domain is deleted from the constant regions. HCAb production does not require an IgM stage for effective pre-B cell signaling, and antigen-specific heavy-chain-only IgGs are produced upon immunization (3). Camelid VHH segments are soluble and this is attributed to the presence of a germ line-encoded tetrad of specific hydrophilic amino acid substitutions at the hydrophobic interface of the conventional VH domain that normally interacts with a variable light chain domain (VL) (6) and a CDR3 loop that folds over the VHH, covering the side of the domain that normally interacts with a VL domain (7).

In contrast, human VH domains usually aggregate and are less stable due to exposure of the hydrophobic amino acids at the former interface (8) and the loss of contacts between the V regions, respectively. This limits their applicability [see Rosenberg (9) and Fahrner et al. (10)]. However, extensive engineering and selection (7, 8) mainly by increasing the hydrophilicity of the VH domain (8) and by replacing exposed hydrophobic residues in the CDR3 region (7) will increase the solubility of the VH domain. These methods have the disadvantage that they require extensive work and that amino acid changes particularly in the CDR3 region could reduce or change the specificity and affinity of antigen binding.

We hypothesized that the mouse would be much more effective at such engineering *in vivo* through the natural process of selection. We, therefore, introduced a fully human HCAb locus into mice to generate fully human HCAbs of different classes or fragments thereof in response to antigen challenge for use as therapeutic agents in man. To this end, we replaced the llama VHH domains with human VH domains in the transgenic construct used by Janssens et al. (3), generated a number of transgenic lines, and derived a number of HCAb against different antigens by hybridoma and phage display technology. Both the hybridoma and phage display technologies have a number of disadvantages, are quite laborious, and in addition phage display needs additional full-format HCAb recloning in eukaryotic systems.

It has been known that long-term production of Abs is maintained by a combination of short-lived and long-lived plasma cells (PCs), usually defined functionally as Ab-secreting cells (ASC). Although short-lived ASC die within 3–5 days, Ab levels can be maintained by continuous proliferation and differentiation of memory B cells (MBC) into short-lived ASC upon continuous reactivation (11, 12), such as persistent antigen exposure. Alternatively, long-term production of Ab is maintained by long-lived ASC, which migrate to survival niches within the bone marrow (13, 14) and spleen (15). Thus, we used CD138^+^ CD45R B220^low/−^ CD19^low/−^ antibody-secreting PCs (16), bone marrow, and spleen of immunized mice containing a human HCAb locus (4HVH) as the enriched RNA source for the production of an expression library.

**Figure 1:**
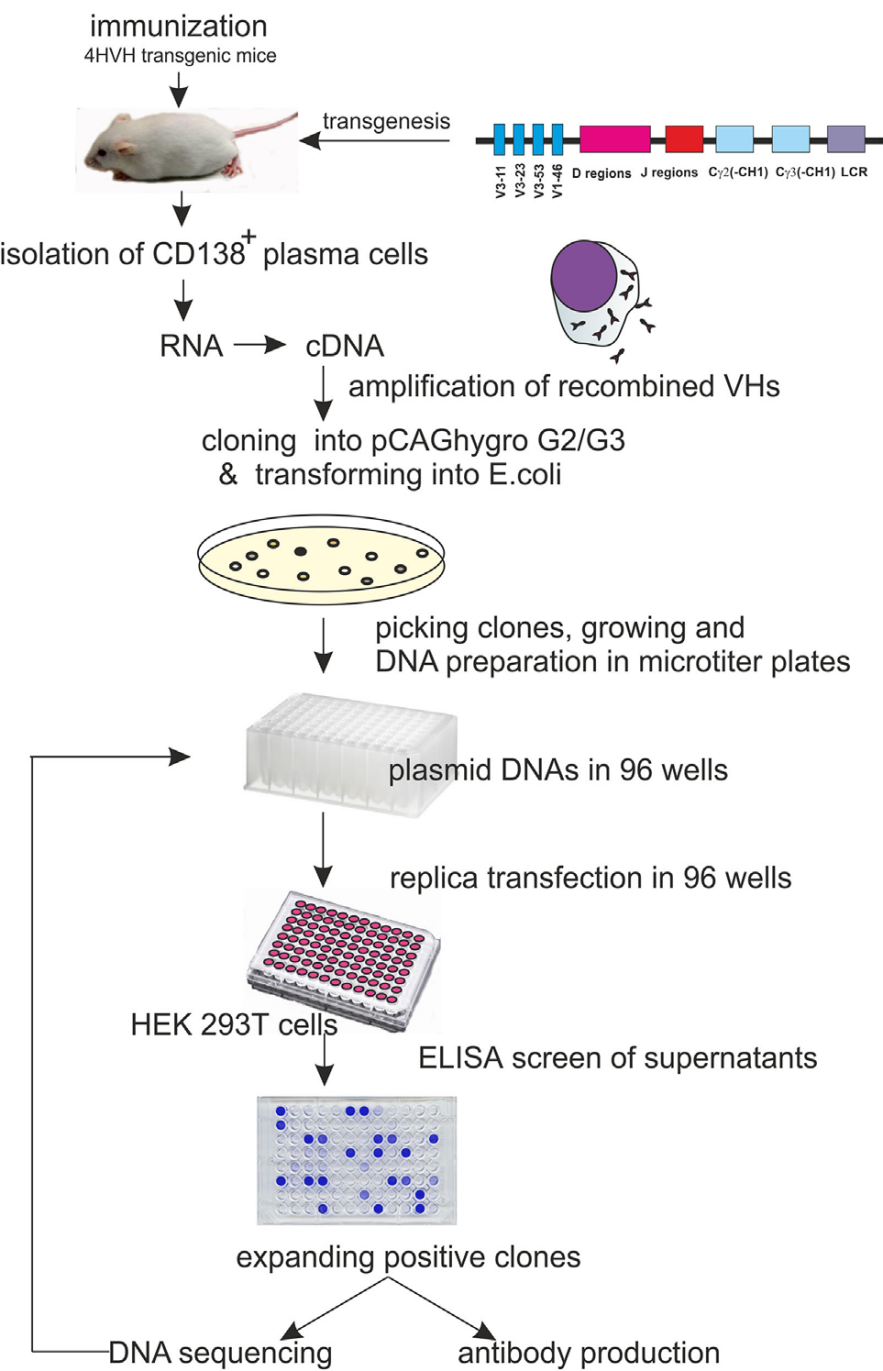
**Schematic representation of the procedure leading to heavy-chain-only antibody (HCAb) production with human HCAb locus construct used for transgenesis**. It carries 4VH regions, all of the human D and J regions and the Cγ2 and Cγ3 regions lacking the CH1 domain.

Here, we describe an automatable alternative method for rapid cloning and identification of antigen-specific HCAbs from immunized transgenic mice (4HVH) carrying a fully human heavy chain locus by cloning the VDJ region of the HCAb cDNA directly into a mammalian expression vector and identifying the human embryonic kidney 293 T (HEK293T) clones secreting antigen-specific HCAb (See Figure 1).

## Methods

### Immunization

4HVH transgenic mice and control wild-type (WT) mice were immunized according to the protocol approved by the Dutch Experimental animal committee DEC Nr EUR 1944. Briefly, mice were injected i.p. five times at 2-week intervals with the influenza virus X-31 hemagglutinin (HA), prepared as described by Ruigrok et al. (17), and dissolved in phosphate-buffered saline (PBS) at pH 7.4 using Stimune adjuvant (Prionics, Switzerland) according to the formulation provided by the supplier. The last injection was without the adjuvant. Four days after the last injection, 4HVH mice were sacrificed and PCs isolated.

### PC Isolation and Library Construction

A single-cell suspension was prepared from spleens and femurs in 0.5% BSA, 2 mM EDTA in PBS. Cells were counted (Burke chamber), and magnetic cell sorting of CD138^+^ cells was performed using mouse CD138^+^ plasma isolation kit (Miltenyi Biotec GmbH, Germany) according to the manufacturer’s instructions. Basically, this consists of two steps: first a depletion of non-PCs by indirect magnetic labeling of CD49b and CD45R cells with a non-PC depletion cocktail and anti-biotin microBeads followed by magnetic separation using LD columns (Miltenyi Biotec GmbH, Germany), and the next step is a positive selection of PCs by direct labeling with CD138 MicroBeads followed by magnetic separation on a MS column (Miltenyi Biotec GmbH, Germany). PCs eluted from the column were spun down and the pellet resuspended in 400 µl of Ultraspec™ RNA reagent (Biotecx laboratories, Inc., Houston, TX, USA). Total RNA was made according to the manufacturer’s instructions. The RNA was dissolved in 20 µl of dH_2_O and 3 µg was used in a 20 µl reaction volume for a first strand cDNA synthesis using SuperScript™ II RT (Invitrogen by Fisher Scientific, USA) according to the instructions using oligo dT priming with oligo (dT) 12-18 primer (Invitrogen by Life Technologies, USA). The transgenic mice contain four different VHs (1-46, 3-11, 3-53, and 3-23), and hence three different leader-specific primers were designed for the 5′-end. All of them contained a *Pvu*II site that is unique in the final expression vectors (pCAG hygro G2 and pCAG hygro G3 containing leader sequence from VH3-23). The following 5′-primers were used:

lib-3-23/53-S: 5′-GTGTCCAGTGTGAGGTGCAGCTG-3′,
lib-3-11-S: 5′-GTGTCCAGTGTCAGGTGCAGCTG-3′, and
lib-1-46-S: 5′-GTGCTCACTCCCAGGTGCAGCTG.

For the 3′-end, we used primer HINGEIgG2rv [previously used and described for phage display library (3)] and HINGEIgG3rv: 5′-AATTGTGTGAGCGGCCGCACCAAGTGGGGTTTTGAGCTC.

An additional 3′-end primer was used: lib-IgG2/3-CH3-AS: 5′-CTGACCTGGTTCTTGGTCATCTCCTC.

This primer from the CH3 constant region is common for both G2 and G3. However, in combination with any of the 5′-primers, it amplifies only IgG2. To make sure that all of the VHs were represented in the library, each of the 5′-end primers was used separately in combination with each of the 3′-end primers, and the products were mixed in an equimolar ratio later. PCR was performed with high-fidelity DNA polymerase Phusion™ (New England Biolabs, Inc., USA) using cycling conditions recommended by the manufacturer for the three-step protocol with an annealing temperature of 68°C and 35 cycles in total. PCR products for IgG2 were cut either with *Pvu*II/*Bst*EII or *Pvu*II/*Bsr*GI (unique site in the constant region) if the amplified fragment originated from using the combination with the Lib-IgG2/3-CH3 primer and cloned into *Pvu*II/*Bst*EII or *Pvu*II/*Bsr*GI cut and phosphatase-treated pCAGhygro G2 vector.

PCR products for IgG3 were digested with *Pvu*II/*Bst*EII or *Pvu*II/*Sac*I and cloned into *Pvu*II/*Bst*EII cut and phosphatase-treated pCAGhygro G2 or *Pvu*II/*Sac*I cut and phosphatase-treated pCAGhygro G3 vector.

**Figure 2:**
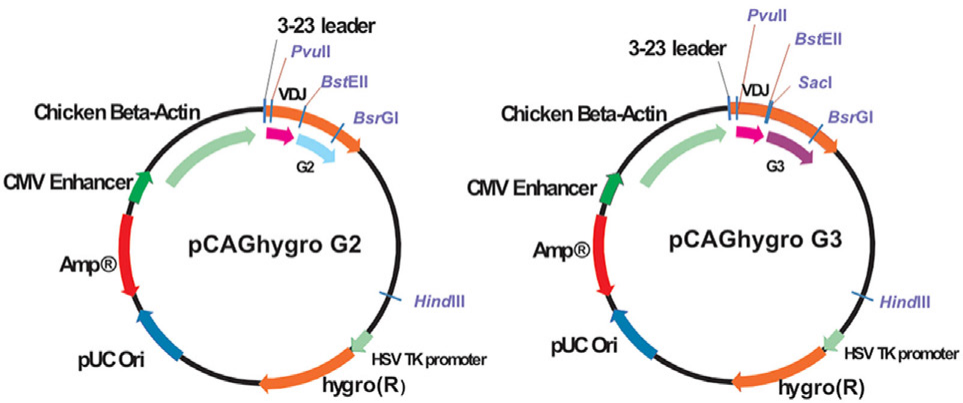
**Schematic representation of the pCAGhygro G2 and the pCAGhygro G3 eukaryotic expression vectors used to generate the HEK293T cell libraries**.

pCAGhygro G2 and pCAG hygro G3 are depicted in Figure 2. In short, the vector contains an ampicillin resistance gene for bacteria selection and a hygromycin resistance for the eukaryotic cell selection. The HCAb expression is driven by ubiquitous CMV enhancer and chicken beta actin promoter. The human VH 3-23 leader is responsible for secretion of the antibody. Constant regions have a deleted CH1 exon.

### Transformation, Plasmid Preparation, and Transfection into HEK 293T Cells

All ligations were done overnight at 16°C in 10 µl total volume using 120 ng of vector alone (control) or in combination with 30 ng of insert using T4DNA ligase (Promega, Madison, WI, USA). Ligations were diluted 5× (40 µl of dH_2_O into 10 µl ligation), and 1 µl was used to transform 20 µl of electrocompetent MegaX DH10B™ T1^®^ cells (Invitrogen by Life Technologies, Grand Island, NY, USA) according to the instructions provided by the manufacturer.

Plating was done on 2XTY/Amp agar plates, and 960 individual colonies were picked into 1.5 ml of 2XTY/Amp medium in 96-well format. A total of 960 DNA plasmid preparations were done using NucleoSpin R96 flash (Macherey-Nagel, Germany) designed for rapid manual and automated 96-well DNA preparation of high- and low-copy plasmid and Bac DNA from *Escherichia coli*. Each DNA was dissolved in 50 µl sterile dH_2_O. A total of 200 ng of DNA (estimated to be in10 µl of DNA preparations based on random sample concentration measurements) was used for transfection into HEK 293T cells. The HEK 293T cells were plated into 96-well plates and transfected using Lipofectamine™ 2000 (Invitrogen, Carlsbad, CA, USA) according to the manufacturer’s instructions for 96-well format. The following day, medium was removed and replaced with DMEM medium (Lonza, Belgium) supplemented with hygromycin (Roche, Diagnostics GmbH, Germany) at a concentration of 200 µg/ml, non-essential amino acids (NEAA, Lonza, Belgium), and 10% fetal calf serum (FCS). On day 4 posttransfection, 100 µl of medium was taken from each well for an antigen-specific enzyme-linked immunosorbent assay (ELISA) and replaced with a fresh medium.

### ELISA Assay

Enzyme-linked immunosorbent assay plates were coated overnight at 4°C with 5 µg/ml of antigen in PBS or PBS only. Blocking was done for 1 h at room temperature (RT) with 1% milk 1% BSA/PBS (W/V). This was done to exclude possible “sticky” binders to plastic or non-specific binders to BSA/milk. Washing steps included 3× PBS/0.05% Tween-20 and 3× PBS. A total of 50 µl of transgenic mouse serum were diluted in PBS (for initial testing of immunized animals), or supernatants from HEK 293T cells were mixed with 50 µl of 2% milk 2% BSA/PBS (W/V) and incubated for 1 h at RT. After washing, the antigen-specific serum/supernatants were detected by incubation with goat antihuman IgG Fc coupled to horse radish peroxidase (HRP; Jackson Immuno Research Laboratories, Inc., USA) diluted 1:5,000 for 1 h at RT, followed by washing steps and incubation with peroxidase substrate BM Blue POD (Roche Diagnostics GmbH, Germany). The reaction was stopped with 1M H_2_SO_4_, and the absorption was measured at 450 nm (against reference wavelength 690 nm).

For the initial testing of immunized WT and 4HVH transgenic mice sera for the presence of antigen-specific mouse antibodies, polyclonal goat antimouse immunoglobulins, HRP (Dako, Denmark) diluted 1:2,000 was used.

### Sequencing

Sequencing of the positive DNA clones was done using primer CAG seq2-s 5′-GCTGGTTATTGTGCTGTCTCATC-3′.

### Initial Affinity Measurement Screen and Full Kinetics

Medium was collected from HEK 293T cells, stably transfected with an HCAb expression vector and grown to confluence. To screen for the clones that expressed the highest affinity HCAb for HA, 200 µl of HEK 293T cell medium from each clone was transferred to a single well of a black 96-well microtiter plate (Greiner Bio-One, Germany). The binding of the HCAbs to influenza HA was studied using the Octet QK (ForteBio, USA). Anti-human IgG-coated tips (ForteBio, USA) were incubated with PBS/0.05% Tween-20 (120 s) to establish baseline signals. To allow the capturing of HCAbs, the tips were then transferred to 200 µl HEK 293T medium containing HCAbs (600 s), HEK 293T medium without HCAbs and PBS/0.05% Tween-20 as a reference. Subsequently, the tips were transferred to 200 µl PBS/0.05% Tween-20 (480 s) to establish the binding levels after the dissociation of non-specific interactions. Next, the loaded tips were transferred to 200 µl 512 nM bromelain-released HA (BHA; 600 s) to allow HA binding to the captured anti-HA HCAbs and to 200 µl PBS/0.05% Tween-20 (1,200 s) to determine the dissociation rate of the HA from the HCAb.

The reference binding signal was subtracted from the binding curves, and the binding data were fitted to a 1:1 binding model using Octet 4.0 software. The HCAb clones that showed binding in initial affinity screen were selected for further analysis.

Full kinetics experiments were performed with purified HCAbs dialyzed against PBS. Anti-human IgG(Fc) sensors were dipped for 180 s in PBS/0.05% Tween 20. HCAb (10 µg/ml) was used for the loading step (300 s, 900 rpm), followed by 180 s in PBS/0.05% Tween 20. The association step with HA ligand in a concentration range from 0 to 512 nM was performed (600 s at 900 rpm), followed by dissociation step of 1,200 s in PBS/005% Tween 20 at 900 rpm. All steps were done in 200 µl volume. The buffer only well (PBS/0.05% Tween 20) was used as a reference well. The reference binding signal was subtracted from the binding curves, and the binding data were fitted to a1:1 binding model using Octet 7.1 software.

### Production and Purification of Anti-HA HCAbs

Positive clones were further expanded in medium containing 10% FCS. Alternatively, plasmid DNA was linearized with *Hin*dIII, stably transfected into HEK293T cells, and individual clones picked for the purpose of selecting the best expressors.

For production purposes, clones were grown in 15 cm Petri dishes in 25 ml of OPTI-MEM^®^ (1×) + GlutaMAX™ (Gibco by Life technologies, CA, USA) medium. Medium was collected and replaced twice per week. Collected medium was spun down at 1,000 rpm (Eppendorf centrifuge 5810R) for 5 min to remove cell debris, and HCAbs were purified on Protein A agarose Fast Flow 50% (V/V; Sigma, USA). A total of 100 µl of Protein A beads were incubated with 50 ml of medium overnight at 4°C on a rotating wheel. After spinning at 1,000 rpm for 5 min, beads were washed in PBS/0.01% Tween-20 and loaded onto homemade columns (insulin syringe with cotton wool), washed 3× with PBS/0.01% Tween-20, 3× with PBS, and eluted with 3M potassium thiocyanate (KSCN). Eluted HCAbs were dialyzed for 5–6 h using Spectra/Por dialysis membrane MWCO 10000 (Spectrum Laboratories Inc., USA) against 1,000× volume excess of PBS at 4°C. The procedure was repeated three times.

### Production and Purification of VH Domains in *E. coli*

VH domains from HCAbs were cloned without a tag into pET SUMO vector (Champion pET SUMO Expression system, Invitrogen, CA, USA). Production of fusion protein, cleavage, and removal of SUMO and SUMO protease were done according to the manufacturer’s instruction with recommended reagents.

VH domains in fusion with SUMO were purified from inclusion bodies starting with 200 ml BL21 transfected cells after 4 h of induction (1 mM IPTG). Cells were harvested, pellet frozen overnight, and lysed in 20 ml lysis buffer (50 mM KPO_4_, pH 7.8; 400 mM NaCl; 10 mM KCl; 10% glycerol; and 0.5% Triton X-100). After sonication 20 × 20 s ampl 12 and spinning for 15 min at 4,000 rpm at 4°C, pellet was taken in 1 ml PBS with addition of 10 µl benzonase for 30 min at RT. After washing in washing buffer (100 mM Tris pH 7.5; 5 mM EDTA; 2 M urea; and 2% Triton X-100), sample was spun for 15 min at 4,000 rpm, washed 2× in 10 ml of 100 mM Tris pH 7.5 and 5 mM EDTA. Pellet was taken into 8 M urea; 10 mM Tris pH 7.5; and 2 mM DTT, rotated for 2 h at RT, spun for 30 min, 15,000 rpm at 4°C, and the supernatant was used in refolding. Base refolding buffer 2 from Pierce refolding kit was used (440 mM 1-arginine; 55 mM Tris pH 8.2; 21 mM NaCl; and 0.88 mM KCl). Protein was diluted to 1 mg/ml; in 8 M urea, 10 mM Tris pH 7.5; and 2 mM DTT. To fold 0.5 mg of protein, we used 9 ml base refolding buffer, 100 µl 0.1 M EDTA, 6.75 mg GSH (reduced glutathione), 2.65 mg of GSSG (oxidized glutathione), and H_2_O up to 9.5 ml. Protein was added in 50 µl aliquots, well mixed, and left on ice for at least 1 min after each addition. After overnight incubation at 4°C, sample was dialyzed against PBS buffer.

### Solubility Test and FPLC

A total of 500 µg of each HCAbs was concentrated to an end point of 30 µl volume using Centriprep-10 K centrifugal filter device (Merck Millipore Ltd., Ireland) as suggested by the manufacturer. Concentrated samples were diluted 10× in 8M guanidine hydrochloride (Gdn-HCl) and the OD at 280 nm measured on a NanoDrop 2000 spectrophotometer (Thermo Scientific, Ireland). The same procedure was used for selected VHs expressed in *E. coli*.

A total of 2 µg of purified and PBS-dialyzed HCAb in 50 µl of PBS was run on a Superdex 200 (3.2/30) column (GE HealthCare Life Sciences, USA) on the FPLC Smart system from Pharmacia. Samples were run in PBS. Bio-Rad’s gel filtration standard as a mixture of molecular weight markers ranging from 1,350 to 670,000 Da was used as a control. The same procedure was done with selected VHs expressed in *E. coli* run on a Superdex 75 (3.2/30) column (GE HealthCare Life Sciences, USA).

### Virus Reduction Assay

The microtiter plaque reduction assay, as described by Matrosovich et al. (18), was performed using MDCK-SIAT1 cells. Twofold dilutions of the HCAbs were incubated on cell monolayers prior to addition of virus. The neutralization titer is determined as the reciprocal of the dilution of HCAbs, which corresponds to 50% reduction in plaque formation, compared to the virus control.

### Hemagglutination Inhibition (HI) Assay

Hemagglutination inhibition assays were performed according to standard methods, Kendall et al. (19), using 0.75% and 1.0% of Turkey and guinea pig red blood cell suspensions, respectively. Four HA units and twofold dilutions of HCAbs were used in these assays. HI titers are reciprocals of the highest dilution of HCAbs, which inhibited hemagglutination.

## Results

4HVH transgenic mouse lines contain four non-mutated human germ line heavy variable regions (VH3-11, VH3-23, VH3-53, and VH1-46) followed by all human D and J regions, the C_γ_2 and C_γ_3 constant regions, each with a deleted CH1 exon and the human immunoglobulin 3′LCR (Figure 1). All transgenic mouse lines rearranged the human HCAb locus and rescued B cell development in a mouse Cμ knockout background (20). They express HCAb dimers in the serum of the correct size (75–90 kDa). These mice were immunized and used as a source of antigen-specific antibodies in developing the method of cloning HCAbs directly into mammalian cells.

### Immunization of Transgenic Mice Leads to Successful Production of Antigen-Specific HCAbs

A total of 20 or 50 µg of influenza X-31 HA protein, prepared as described by Ruigrok et al. (17), per mouse was injected intraperitoneally in 2 weeks intervals into eight of 4HVH transgenic mice (two transgenic lines, 4HVH-B and 4HVH-C, originating from different founders, having different integration site of the transgene) and two WT mice as a control, using Stimune as an adjuvant. After the third injection, mice were bled, and an ELISA assay was performed on serum, using HA protein-coated plates (5 µg/ml) and anti-human IgG-HRP or anti-mouse IgG-HRP for the detection of HA-specific antibodies. In seven out of eight transgenic mice and two out of two WT mice, HA-specific antibodies were detected (Figure S1 in Supplementary Material). The higher amount of antigen did not lead to a better response, thus we concluded that 20 µg is sufficient for successful immunization. Seven transgenic ELISA positive mice were immunized three or more times with 20 µg of HA.

### HEK 293T HCAbs Library Construction and Subsequent Screening Result in a Diverse Repertoire of Antigen-Specific Soluble Antibodies

Four days after the last injection without adjuvant, mice were sacrificed, and CD 138^+^ PCs were isolated from bone marrow and spleen. Total RNA was isolated, and cDNA was synthesized (depicted schematically in Figure 1). Human VDJ domains were amplified using a set of three different 5′-end primers specific for the leader sequences in the transgenic mouse construct in combination with two different 3′-end primers specific for either the human IgG2 or IgG3 hinge (3). Due to the high sequence similarity, the same 5′-end primer was used for amplification of both VH3-23 and VH3-53. All 5′-primers were designed to contain a *Pvu*II restriction site. A *Pvu*II site appears usually at the beginning of the DNA encoding VH regions (third to fourth codons at the amino acid level) and rarely occurs anywhere else in human VHs. Amplified PCR products were cut either with *Pvu*II/*Sac*I or with *Pvu*II/*Bst*EII restriction enzymes. The *Sac*I site is a unique site at the beginning of the IgG3 hinge, while the *Bst*EII site in frame 4 is unique in most of the VHs and is commonly used for constructing phage libraries (21, 22).

**Figure 3:**
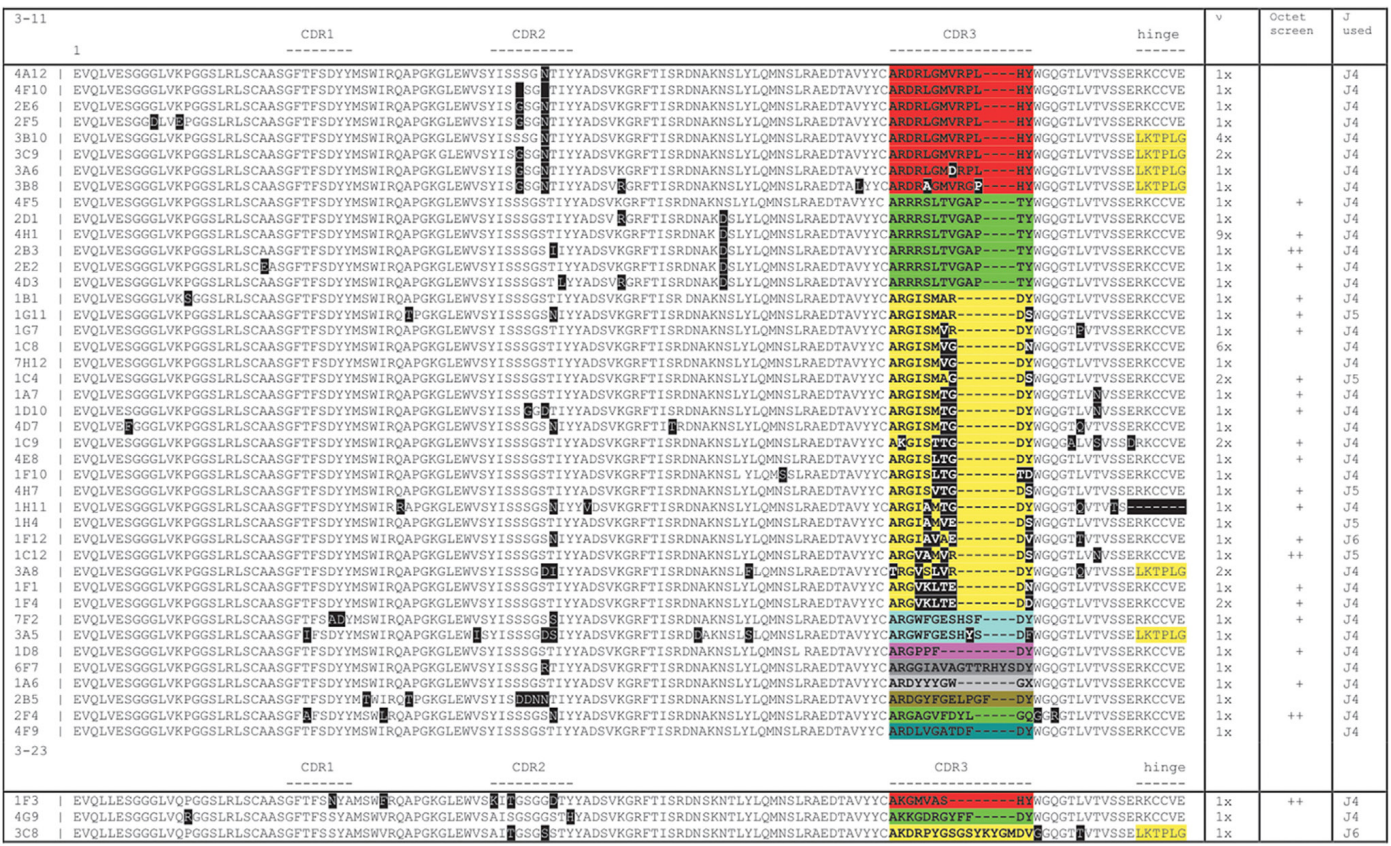
**Sequence analysis of hemagglutinin X-31 ELISA positive clones showing a broad range of diversity based on somatic mutations (black boxes) and CDR3 loops (blocks of different colors)**. The columns on the right show the number of times a particular sequence was found, whether it tested positively on Octet and which J region was used. Yellow shading at the carboxy-terminal end of the sequence shows an IgG3 sequence (LKTPLG), the others are IgG2 (RKCCVE).

The VDJ fragments were cloned into bacteria using either pCAGhygro G2 or pCAGhygro G3 expression vectors, containing the ubiquitously expressed chicken β-actin promoter, the leader sequence from human VH3-23, and the constant region of human IgG2 or IgG3 (Figure 2). The ligated cDNA was transfected into electrocompetent *E. coli* cells, totaling 1 part out of almost 700,000 of the available RNA in the mice (PCs were isolated from half of the total number of cells, 1/4 of total RNA was made into cDNA, 1/30 of the gel purified PCR amplified cDNA was used in the ligations; 1/50 of the ligation was transformed, 1/32 of the transformed bacteria was plated, and 0.57 of total colonies counted were picked from those plates). A total of 960 colonies were picked from both the G2 and G3 library into 96-well plates filled with 2XTY medium. The resulting recombinant bacteria were grown overnight, and plasmid DNA was prepared in the same 96-well format. In parallel, HEK 293T cells were also grown in a 96-well format and transfected with the plasmid DNA maintaining the same 96-well format. A 96-well HA-specific ELISA screen was performed with supernatants from each well 4 days after transfection. This yielded 66 positive supernatants/clones. The corresponding cDNAs were sequenced (Figure 3), which showed that two out of the four available variable segments were used preferentially (VH3-11 and VH3-23); the VDJ domains contained somatic hypermutations; J4 was predominantly used but J5 and J6 were also found; both IgG2 and IgG3 antibodies were present, confirming that class switching occurred in the transgenic mouse. Most prevalent are IgG2 HCAbs, and it is not surprising taking into account that the IgG2 constant region is the most proximal in the transgenic construct and thus the first to recombine. Out of 66 sequences, 45 were unique and these account for 33 different DJ regions. Based on the different CDR3 regions, the HCAbs were classified into 13 distinct groups represented in different colors (Figure 3). Positive clones were cultured further, and supernatants were collected for affinity screening, using the same HA antigen preparation. Twenty four clones showed significant binding. A selected number of clones (based on sequence diversity and affinity) were produced in serum free medium. The antibodies with low affinities were not tested at all in a functional assay (see below). Four groups represented by 7F2 (Figure 3, blue), 2F4 (green), 1F3 (red), and 3A8 (yellow) were left. Of these, 1F3 and 2F4 appear to bind the same epitope(s), while 3A8 and 7F2 appear to bind a different but overlapping epitope(s).

### Characterization of Anti-HA Antibodies

**Figure 4:**
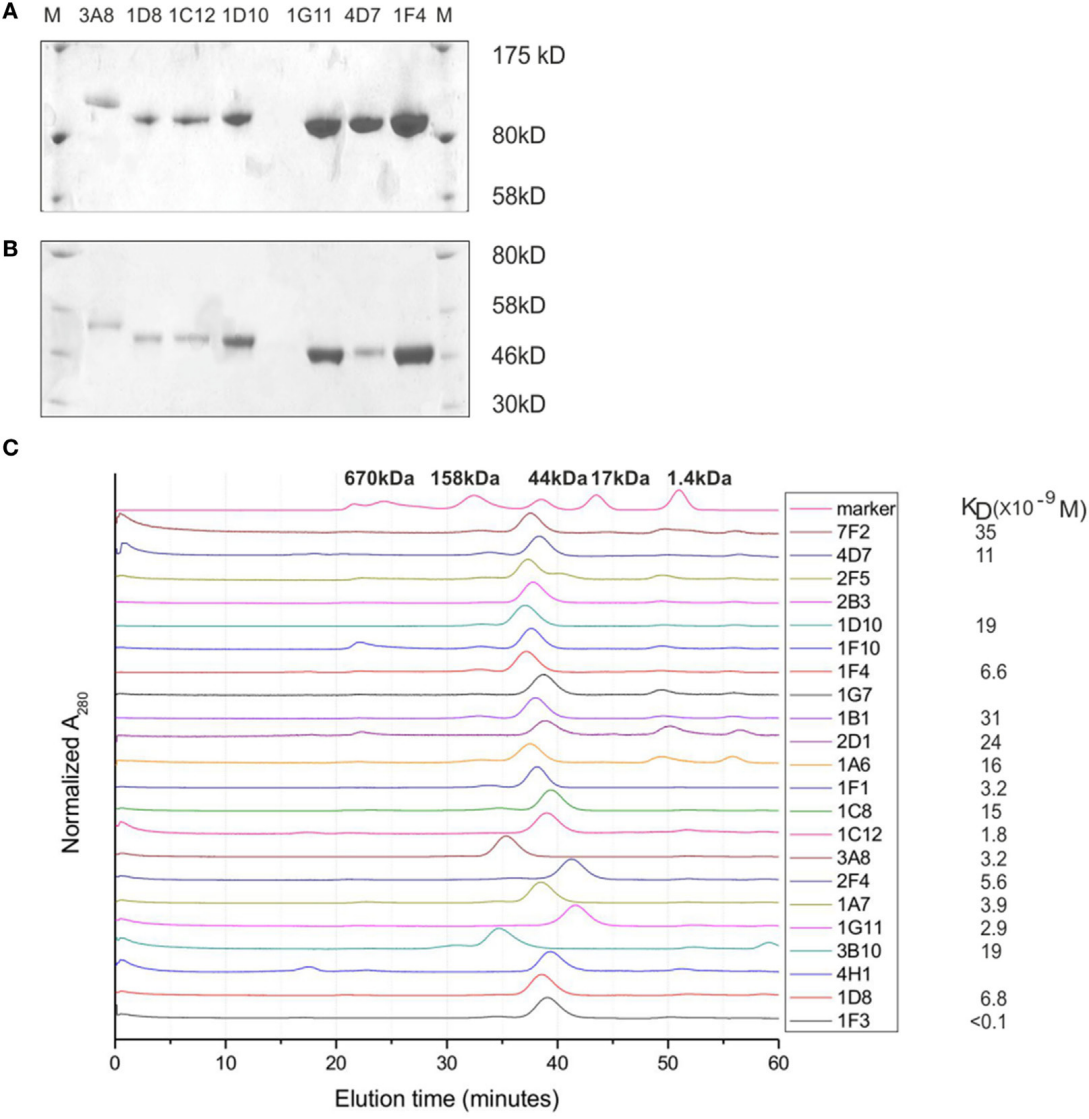
**Characterization of heavy-chain-only antibodies (HCAbs)**. **(A,B)** An example of SDS-PAGE gels run under non-reducing conditions **(A)** and reducing conditions **(B)** shows that HCAbs form dimers. **(C)** HPLC SMART profiles show a single peak for each antibody, the solubility and affinity measurements for selected HCAb clones. Note that 3A8 and 3B10 are IgG3 HCAbs and are of higher molecular weight due to a longer hinge region.

Heavy-chain-only antibodies from medium were purified on protein A, eluted in 3M KSCN, and dialyzed against PBS. The average yield was estimated to be 2–4 µg/ml of medium. On SDS-PAGE gel, HCAbs are of expected size for a monomer (~40–45 kDa) under reducing and of a dimer (~80–90 kDa) under non-reducing conditions (Figure 4A). Size differences originate from different sizes and compositions of VH regions and different sizes (longer hinge) of IgG3 HCAbs, accounting for >5 kDa difference per monomer in comparison to IgG2 HCAbs. The profiles on size chromatography (smart columns) showed a single peak of the expected size; the peaks of the IgG3 HCAbs, 3A11, and 3B10 were of higher molecular weight than IgG2 HCAbs (Figure 4B). Solubility in PBS was tested with 500 µg of each HCAb by concentrating it to ~30 µl of final volume. The selected HCAb differed with concentrations up to 15.4 µg/µl for 1F1, the equivalent of 30 mg/ml of a normal H2L2 antibody (Figure 4C). Further concentration of the very soluble antibodies was not tested. Two of the VHDJ segments (3A8 and 2F4) were also expressed in bacteria and shown to be soluble in at least 5 mg/ml, which is also equivalent to 30 mg/ml of a normal H2L2 antibody (Figure S2 in Supplementary Material). Binding affinities of the HCAb were determined on an Octet instrument with purified antibodies. A BHA preparation (23) that lacks a transmembrane anchor was used to prevent rosette formation and aggregation of antigen. The majority of KD values (9 of 17) for HCAbs were in the 10^−9^ molar range, 7 were in the 10^−8^ molar range, while the KD value for the highest affinity 1F3 HCAb was better than 10^−10^. HCAbs 1G7, 1G11, 2F4, 1F4, 1C12, 4H1, and 3A11 react on Western blots under both non-reducing and reducing conditions recognizing denatured BHA monomer and the BHA1 chain, respectively. 7F2 reacts with BHA only under non-reducing conditions, while 1F3 preferentially recognizes BHA1 under reducing conditions (data not shown).

### Anti-HA HCAbs Function in Virus Neutralization and HI

**Figure 5:**
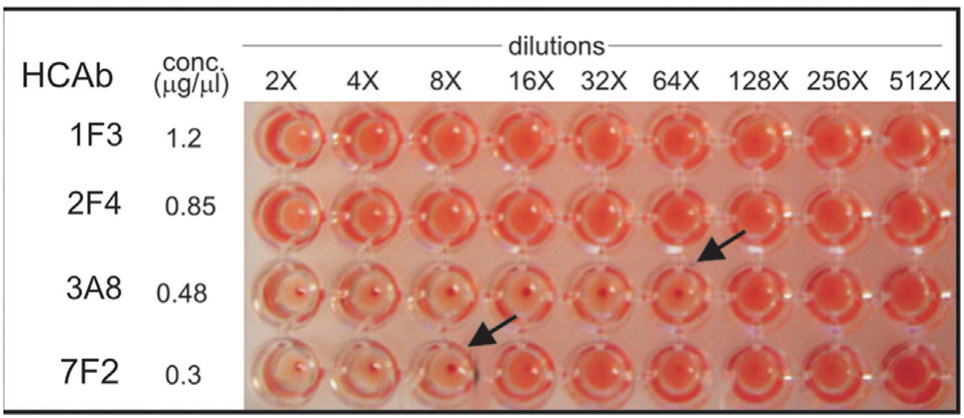
**Hemagglutination inhibition assay performed on Turkey red blood cells**. 3A8 and 7F2 HCAbs inhibit at a concentration of 0.0075 mg/ml (84 nM) and 0.0375 mg/ml (483 nM), respectively.

The HA HCAbs were first evaluated for their ability to neutralize X.31 (H3N2) influenza virus in a plaque reduction assay. Neutralization is reported as the reciprocal of the highest dilution of the antibodies corresponding to 50% plaque reduction compared to the virus control. A total of 18 of the antibodies tested show visible inhibition, and 50% reduction is observed for four HCAb, 1F3 > 2F4 > 3A8 > 4H1 = 7F2 (Table 1). A modified neutralization experiment was also performed, where antibodies were incubated for 30 min with the virus prior to addition to MDCK-SIAT1 cells. This led to an increased neutralizing effect especially in the case of 2F4 and 1F3 antibodies [2F4 neutralizing at ≥1.4 µg/ml (18nM) and 1F3 at ≥0.9 µg/ml (11.7nM)]. Ferret anti X-31 serum and HC19, an anti-X31 HA mouse monoclonal antibody, were used as positive controls. 3A8 and 7F2 HCAbs also showed an HI performed with Turkey red blood cells (Figure 5) and with guinea pig blood (data not shown), suggesting that 3A8 and 7F2 neutralize infectivity by obstructing the binding of virus to the host cell.

**Table 1:** **Neutralization analysis of heavy-chain-only antibodies (HCAbs) against X-31 influenza virus**.

	NI	I	NI	I	NI	I	NI	I	NI	NI	NI
HCAbs	2F4	2F4	1F3	1F3	3A8	3A8	4H1	4H1	7F2	HC19	X31
Concentration (mg/ml)	0.85	0.9	1.2	1.2	0.48	0.55	1.6	0.8	0.4	NK	NK
X31(50% reduction)	32	640	>256	>1,280	16	20	2	2	2	6,400	2,560
X31 (any visible reduction)	64	>1,280	>256	>1,280	64	160	2	8	64	>12,800	>5,120

*The numbers show best neutralizing capacity for 1F3 HCAb at ≥0.9 *μ*g/ml (~11nM), followed by 2F4 at 1.4 µg/ml (~18nM)*.

*NI, not preincubated with the virus; I, preincubated with the virus; NK, not known; neutralization titer, reciprocal of the 50% plaque reduction*.

## Discussion

Here, we describe a very efficient method for obtaining fully human, antigen-specific, soluble, high-affinity HCAbs, which can easily be automated. Our approach is based on capturing the antibody repertoire of antibody-secreting PCs from both bone marrow and spleen of immunized transgenic mice. Recently, other laboratories have used mouse spleen PCs (24), mouse bone marrow PCs (25), human peripheral blood PCs (26, 27), or human MBC from patient recovered from infection (28) to obtain antigen-specific monoclonal antibodies. Basically, those methods utilize single-cell sorting, single-cell RT-PCR, and natural VH–VL pairing (27, 28). Screening methods have been developed to improve efficiency and enable detection of antigen-specific secreting cells before a single-cell RT-PCR step, such as enzyme-linked immunospot (29), immunospot array assay on chip or microengraving (30). Recently, a method was described (24) omitting a screening step, utilizing massive DNA sequencing and bioinformatics tools to analyze the VL and VH gene repertoires and to find several abundant VH and VL sequences that are paired based on their relative frequencies with 78% efficiency and a method that combines the next-generation sequencing and protein mass spectroscopy to obtain antigen-specific antibody repertoires (31). DeKosky and colleagues developed a low-cost, single-cell, emulsion-based technology for sequencing of antibody VH–VL repertoires with even better pairing precision of >97% (32).

The 4HVH transgenic mice produce human HCAb, without light chains, thus there is no pairing or the necessity for single-cell RT-PCR. The major concern was the solubility of human VH domains, that are not soluble *per se*, but as our results show, antigen-specific HCAbs selected through our screen show high solubility both as a full-length HCAbs and as VH fragments only, thus, the method allows easy isolation of soluble VH regions and the construction of multivalent soluble VH complexes.

The soluble human VH domains obtained from transgenic mice presented here do not possess the hallmark amino acid changes present in VHHs of camelid HCAbs (at positions 44, 45, and 47), which reduce the hydrophobicity of the former light chain interface. They remain as germ line VH being G, L, and W, respectively. As for replacing the V 37 of VH by more hydrophilic F or Y in VHHs, 1 out of 66 VH domains have mutated at this position to F 37, one to L 37. All V3-11-derived HCAbs have I-37 as in the germ line. We have analyzed many sequences from different immunizations with different antigens, beyond the restricted list of anti HA HCAbs shown in Figure 3. We have seen FR2 substitutions, which are not found in antibodies that comprise heavy and light chain. We found an increased net hydrophobicity within CDR1 and an increased number of charged amino acids present in CDR3, amino acid substitutions within the framework β-pleated sheet leading to increased net hydrophobicity within FR1, and increased number of charged amino acids present in FR3, all of which could lead to solubility of autonomous soluble VH domains obtained from transgenic mice (33).

This experiment was done in 4HVH transgenic mice bred into our own Cμ MT heavy chain knockout background. It was previously reported that Cμ MT knockout mice can produce low levels of IgG antibodies after prolonged time (34). In such a case, mouse light chains might theoretically attach to the human VHs and affect their solubility, which could cause a problem in using hybridoma fusions. If the reason for increased solubility is light chain attachment, the solubility will be hampered by cloning the heavy chain only (VH) in expression vectors. Once produced, such antibodies would have aggregation problems, which we do not see using HEK 293T libraries. Knocking out the loci completely would avoid the problem.

The choice of VHs in the transgenic construct was based on the VH usage in a human population. It is also known that soluble llama VHHs mostly resemble family three of human VHs (6). The fact that in this particular experiment most of the obtained antibodies originated from VH3-11, a few fromVH3-23, while none from V3-53 and VH1-47 could be explained by antigen-related specific usage. A new class of influenza-neutralizing antibodies that target a conserved site in the HA stem, most of them being VH 1-69, have been described, but this VH is not present in our transgenic mouse (35). In other experiments, the other VH regions are also used (data not shown). The soluble human VH domains obtained from transgenic mice presented here do not possess hallmark amino acid changes present in VHHs of camelid HCAbs (at positions 44, 45 and 47), which function to reduce hydrophobicity of the former light chain interface. They remain as in germ line VH situation being G, L, and W, respectively.

The four human VHs obtained from transgenic mice were cloned into human IgG2 and IgG3 vectors to reproduce characteristics of antibodies circulating in transgenic mouse. For most therapeutic purposes, human IgG1 and IgG4 antibodies are preferred, due to their effector functions. Once obtained, selected VHs could be cloned and expressed as human IgG1 or IgG4 in appropriate vectors.

We are improving our transgenic mouse platform by increasing the numbers of germ line VHs in the construct, thus increasing the repertoire. The human constant regions in the new generation of transgenic mice have been replaced by the mouse constant region/s reported to be better suited to the mouse machinery (36), while direct cloning might be performed into human IgG vectors of choice.

The pilot experiment performed with 960 plasmid DNAs from the bacterial library and an estimated 7% vector contamination (plasmids without the cDNA insert) gave 66 ELISA-positive clones, 45 of these being unique. If we take into account that the mice carry only 4VH regions, that only a tiny fraction of the available material was used, and that 83% of retrieved antibody sequences were represented only once in the sample tested, a very high efficiency of obtaining many more high-affinity antibodies in an industrial scale automated/robotized process at low cost can be predicted.

## Ethics Statement

The animal study described was approved by the Animal ethical comity [**D**ieren**e**xperimenten**c**ommissie (DEC)] under the immunization protocol EUR 1944.

## Author Contributions

DD, FG, and JS contributed to the design of the experiments. DD, EB, RJ, RR, JK, and JS contributed to acquisition and analysis of data. DD, FG, and JS contributed to interpretation of data and writing of the manuscript.

## Conflict of Interest Statement

The authors declare that the research was conducted in the absence of any commercial or financial relationships that could be construed as a potential conflict of interest.
