# Supplementary material for: Expression Cloning and Production of Human Heavy-Chain-Only Antibodies from Murine Transgenic Plasma Cells
Source: Front Immunol. 2016 Dec 19;7:619. doi: 10.3389/fimmu.2016.00619 (PMC5165034; doi:10.3389/fimmu.2016.00619)
Supplement: Supplementary file 1 [file Image_1.PDF]

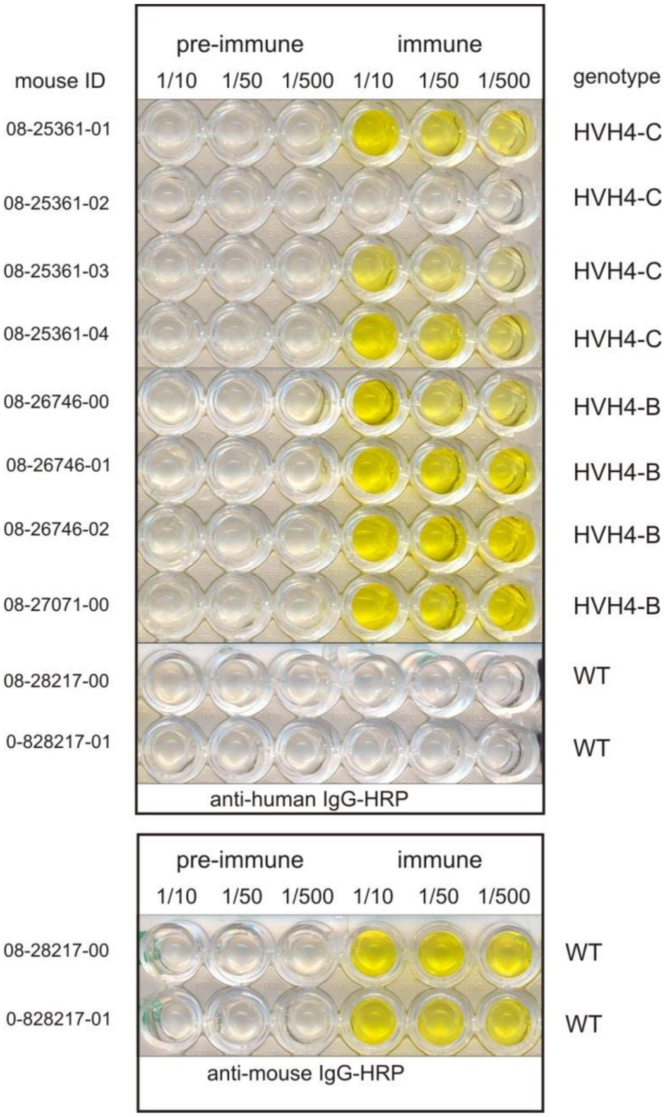

Supplementary Figure S1. Elisa test perform on blood of immunized animals after second boost. All mice except one produce antigen specific antibody. They are detected by anti human IgG antibody in transgenic mice and anti mouse IgG antibody in the wild type (WT) mouse used as a control.
