# Supplementary material for: Expression Cloning and Production of Human Heavy-Chain-Only Antibodies from Murine Transgenic Plasma Cells
Source: Front Immunol. 2016 Dec 19;7:619. doi: 10.3389/fimmu.2016.00619 (PMC5165034; doi:10.3389/fimmu.2016.00619)
Supplement: Supplementary file 2 [file Image_2.PDF]

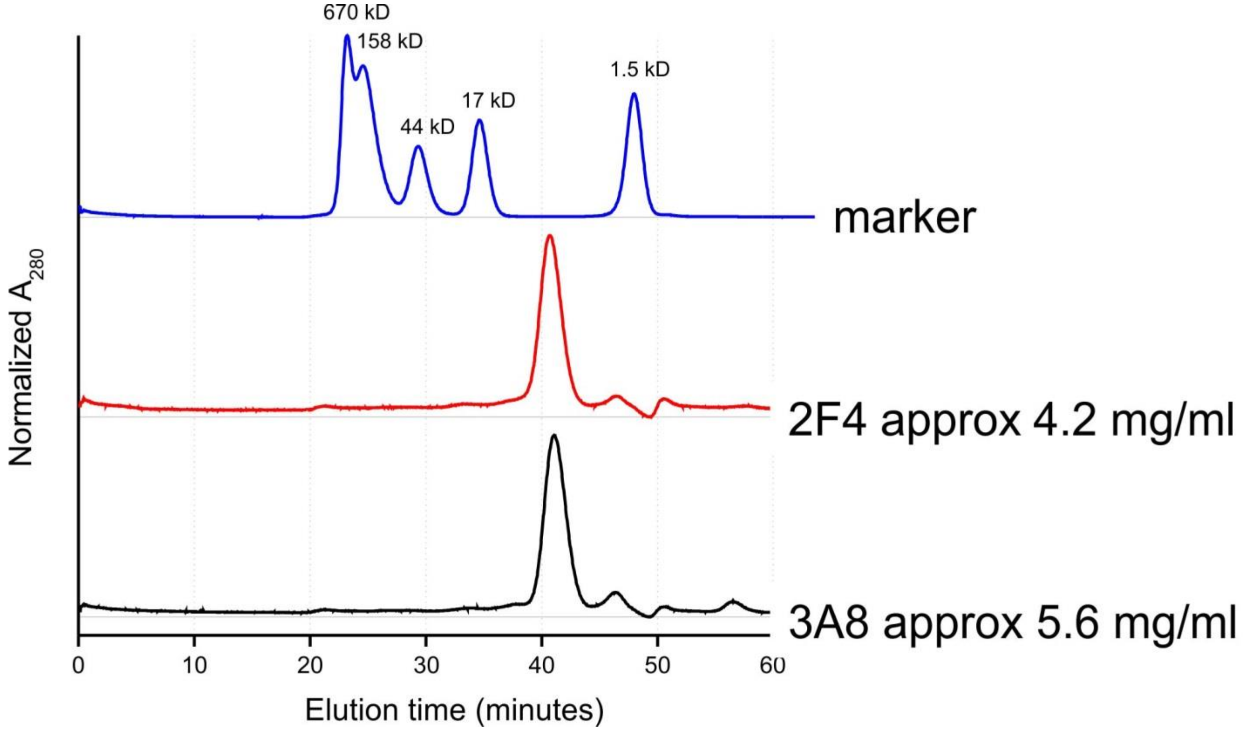

Supplementary Figure S2. VH domains expressed in E. coli SUMO system FPLC (SMART profiles), showing no aggregation.

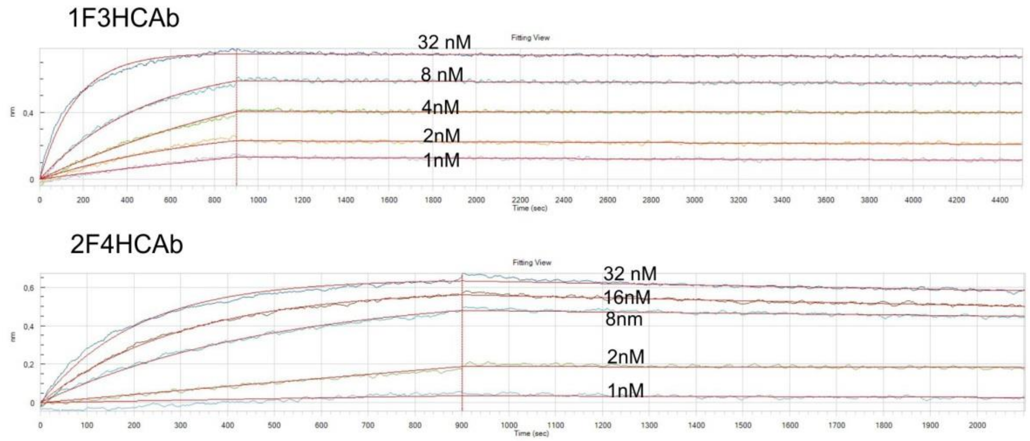

Supplementary Figure S2 . Full kinetics measurement on 1F3HCAb and 2F4HCAb using ForteBio Octet showing high avidities and low dissociation rate. HCAbs were immobilized on anti human Fc sensors. HA antigen was used as an analyte in solution with concentrations ranging from 32nM to 0.
